# 72998 Qualitative analysis of the Los Angeles barbershop study intervention

**DOI:** 10.1017/cts.2021.599

**Published:** 2021-03-30

**Authors:** Joseph Ebinger, Noel Barragan, Ciantel Blyler, Mohamad Rashid, Florian Rader, Moira Inkelas, Tony Kuo

**Affiliations:** 1 Cedars-Sinai Medical Center; 2 Los Angeles Department of Public Health; 3 University of California - Los Angeles

## Abstract

ABSTRACT IMPACT: This translational study demonstrates a method for identifying possible mechanisms underlying a highly effective randomized control trial intervention so that a university-public health agency partnership might replicate intervention components in a scalable, feasible, community version of the program. OBJECTIVES/GOALS: The Barbershop Study was a cluster randomized control trial which demonstrated that clinical pharmacist directed care, provided to African American men in community barbershops, significantly improve hypertension control. We sought to understand which components of the intervention the participants and implementers considered most important. METHODS/STUDY POPULATION: Enrollment in the Barbershop Study included 319 men from 52 barbershops across Los Angeles. Two specialty trained clinical pharmacists led the intervention. We performed 32 structured interviews of 20 study participants, 10 barbers, and 2 clinical pharmacists approximately 1 year after the study’s completion. Interviews consisted of 27, 24 and 19 questions for barbers, participants, and pharmacists, respectively. Interviewees were asked about their experience in the study, barriers and facilitators to participation, effective aspects of the intervention, and less helpful components of the design. Interviews were recorded performed by a research assistant uninvolved in the study. Recordings were then transcribed for a qualitative thematic analysis. RESULTS/ANTICIPATED RESULTS: We anticipate facilitators of participant engagement to include the provision of care in the community and integration of services into a regular task (getting their hair cut). Based on prior conceptual models, we also anticipate the provision of care in a trusted setting to be an effective means to enhance participants’ willingness to follow clinical instructions. An anticipated barrier for participants includes the need to go to an offsite pharmacy to pick up their medications. For barbers, we anticipate themes including a desire to help their community, while barriers include potential decreased productivity due to time spent counseling participants. Pharmacists are expected to identify an enhanced sense of importance in their work, while identifying the need to travel as a barrier to the intervention. DISCUSSION/SIGNIFICANCE OF FINDINGS: Insights from this qualitative analysis may assist with adaptation of the highly effective Barbershop intervention, allowing it to be rolled out at scale. If done successfully, achieved reductions in blood pressure may result in reduced health disparities and prevent thousands of strokes, heart attacks and deaths.

